# miR-24 Attenuates IL-1β-Induced IL-6 Upregulation in Temporomandibular Joint Synovium-Derived Mesenchymal Stem Cells

**DOI:** 10.3390/cimb48101025

**Published:** 2026-10-03

**Authors:** Jiadong Sun, Wenting Liao, Farong Ou, Zhiguang Zhang, Yangpeng Sun, Yiqing He

**Affiliations:** Guangdong Provincial Key Laboratory of Stomatology, Hospital of Stomatology, Guanghua School of Stomatology, Sun Yat-sen University, No. 56 Lingyuan West Road, Guangzhou 510055, China; sunjd5@mail.sysu.edu.cn (J.S.); liaowt9@mail.sysu.edu.cn (W.L.); oufarong3@mail.sysu.edu.cn (F.O.); drzhangzg@163.com (Z.Z.)

**Keywords:** temporomandibular joint, IL-6, miR-24, microtubule affinity regulating kinase 4

## Abstract

Osteoarthritis (OA) is a degenerative joint disease characterized by cartilage degradation and inflammation. The role of microRNA-24 (miR-24) in the development of temporomandibular joint (TMJ) OA remains unclear. To address this, the ability of miR-24 to attenuate inflammatory responses induced by interleukin-1β (IL-1β) in synovium-derived mesenchymal stem cells (SMSCs) by targeting microtubule affinity regulating kinase 4 (MARK4) was investigated. Exploratory analyses of synovial tissues from eight patients with TMJ OA showed a positive association between IL-1β and IL-6 and an inverse association between miR-24 and IL-1β. Overexpression of miR-24 inhibited IL-6 upregulation and nuclear factor-κB (NF-κB) pathway activation induced by IL-1β. Bioinformatics analysis and dual-luciferase assays identified MARK4 as a direct target of miR-24. The knockdown of MARK4 inhibited IL-6 upregulation of SMSCs induced by IL-1β. In a preclinical rat model of TMJ OA, intra-articular delivery of miR-24 agomir was associated with changes in inflammatory and cartilage-related gene expression. These results support a role for the miR-24–MARK4 axis in modulating inflammatory responses in SMSCs and provide a basis for further preclinical investigation.

## 1. Introduction

Temporomandibular joint osteoarthritis (TMJ OA) is a multifactorial condition characterized by progressive cartilage destruction [1]. Patients with TMJ OA commonly experience local pain, limited mouth opening, and maxillofacial deformity, resulting in a significant socioeconomic burden. Repairing damaged cartilage has become a dilemma when treating OA due to its limited self-healing ability [2]. Mesenchymal stem cell-based tissue engineering presents a promising new approach to repairing articular cartilage. Studies have shown that synovium-derived mesenchymal stem cells (SMSCs) possess a more significant chondrogenic advantage, making them suitable for cartilage repair [3].

The chondrogenesis of SMSCs, however, is inevitably affected by the joint microenvironment, including factors such as inflammation, biomechanical forces, and the extracellular matrix [4,5,6]. Synovial inflammation has been associated with joint pain, increased production of inflammatory mediators, and alterations in the intra-articular microenvironment. Recent studies further suggest that intra-articular interventions may modulate the local inflammatory milieu of the TMJ [7,8]. IL-1β and its downstream cytokine IL-6 are significantly upregulated in TMJ OA and have been proven to inhibit the chondrogenic differentiation potential of mesenchymal stem cells [9,10]. Thus, exploring the underlying regulatory mechanism of inflammation response in SMSCs could be valuable for their application in cartilage repair.

Epigenetics focuses on studying phenotypic changes caused by external factors rather than DNA sequence alterations. microRNAs (miRNAs), consisting of 19–25 nucleotides, represent one of the essential epigenetic mechanisms. A growing body of research suggests that miRNAs are broadly involved in various physiological processes, including apoptosis, proliferation, differentiation, and inflammation [11]. Researchers have found that the downregulation of miR-101 inhibits extracellular matrix degradation induced by IL-1β [12]. miR-210 has been shown to inhibit the NF-κB pathway by targeting death receptor-6, thus suppressing the OA inflammatory response [13]. Studies have shown that circular RNA circ_0020014 can regulate IL-1β-induced chondrocyte injury by sponging miR-24 [14]. However, the effect of miRNAs on the inflammatory response in SMSCs has not been fully illustrated. A recent study showed that miR-24 is negatively associated with cartilage impairment and can promote chondrogenesis [15]. However, the impact of miR-24 on the inflammatory activation of SMSCs remains largely unknown.

Using miRNA target-prediction algorithms, miR-24 has been found to potentially regulate microtubule affinity regulating kinase 4 (MARK4) expression. Studies have demonstrated that miR-24 and MARK4 can influence the inflammatory response through the NLR family pyrin domain-containing protein 3 (NLRP3) pathway [16,17]. As a member of the MARK family, MARK4 is known for its ability to phosphorylate microtubule-associated proteins, thereby influencing microtubule dynamics [18]. Furthermore, research has revealed that MARK4 enhances IL-6 secretion by activating the NF-κB signaling pathway in adipocytes [19]. Furthermore, our previous study showed that MARK4 knockdown could inhibit IL-6 upregulation induced by IL-1β by blocking the NF-κB pathway [20]. Research has also found that NF-κB pathway activation contributes to the upregulation of IL-6 in SMSCs inflamed by IL-1β in the TMJ [21]. It was thus hypothesized that the miR-24-MARK4 axis may also be involved in the regulation of inflammation in SMSCs.

In this study, we have investigated the effects and potential mechanism of miR-24 on the inflammation of SMSCs induced by IL-1β. The findings provide insights into the role of miR-24 in the inflammatory activation of SMSCs and offer a foundation for further exploration of its potential as a therapeutic target in cartilage repair and OA treatment.

## 2. Materials and Methods

### 2.1. Acquisition of Synovial Tissues

The human cohort comprised eight surgically obtained TMJ OA specimens available during the study period. No formal power calculation was performed for this exploratory analysis of human tissues; therefore, the correlation analyses were considered exploratory. The specimens were obtained from eight patients with radiographic evidence of TMJ OA who experienced pain or limited mouth opening that was unresponsive to conservative treatment and subsequently underwent surgical treatment at the Hospital of Stomatology, Sun Yat-sen University. The exclusion criteria were malignant tumors, septic arthritis, and intra-articular steroid use. The eight donors had a mean age of 36 years (range, 18–65 years) and were classified as Wilkes stage IV or V. Individual demographic characteristics, surgical sides, imaging findings, and relevant medical histories are provided in Supplementary Table S23. All patients provided informed consent, and basic information was collected. The recorded sampling site was the synovial reflection within the joint cavity. Part of the obtained specimen was used to culture SMSCs, and the remainder was used for RNA extraction. Cell-based experiments were performed using three independently prepared biological replicates. Each biological replicate was generated from an independently prepared SMSC pool derived from at least two donors. Technical replicate wells were averaged within each biological experiment and were not treated as independent biological observations.

### 2.2. Cell Culture

Synovial tissues from patients with OA were washed three times with phosphate-buffered saline (PBS; Gibco, Grand Island, NY, USA) and then trimmed into 1 mm × 1 mm pieces. The tissue was then lysed using 4 mg/mL type I collagenase (Sigma-Aldrich, St. Louis, MO, USA) for 2 h at 37 °C. Upon completion of the digestion process, the precipitate obtained by centrifugation was inoculated into a culture medium containing 10% fetal bovine serum (Gibco) and Dulbecco’s modified Eagle’s medium (DMEM; Gibco). This mixture was incubated for 3–5 days. Subsequently, the SMSCs were maintained at 37 °C in a 5% CO_2_ atmosphere for further experiments and utilized at passages 3–5.

### 2.3. Reagent Preparation

IL-1β (Peprotech, Cranbury, NJ, USA) was dissolved in sterile water containing 0.1% BSA to a concentration of 10 ng/µL. miR-24 mimic, miR-24 inhibitor, mimic negative controls (NCm), and inhibitor negative controls (NCi) (Ribobio, Guangzhou, China) were diluted to 20 μM with diethyl pyrocarbonate water (Gibco). miR-24 agomir and negative control (NC) were diluted to 50 nM with diethyl pyrocarbonate water.

### 2.4. IL-1β Treatment

SMSCs treated with 1, 5 and 10 ng/mL final concentrations of IL-1β for 6 h and treated with a final concentration of 10 ng/mL for 2, 4 or 6 h were collected for further experiments.

### 2.5. Lentivirus Transfection

Lentiviruses either carrying the green fluorescent protein (GFP) empty vector or downregulating MARK4 (siMARK4) were generated by GeneChem Co., Ltd. in Shanghai, China. SMSCs were initially plated at a density of 100,000 cells per well in 6-well plates and allowed to adhere overnight. Subsequently, 20 µL of a viral solution with a concentration of 1 × 10^8^ TU/mL was added to each well. To facilitate transduction, 5 μg/mL of polybrene was added to the medium. After 12 h, the medium was changed to fresh complete culture medium, and 48 h later, the SMSCs were subjected to selection using 1 μg/mL puromycin. Finally, the selected cells were sorted by flow cytometry to achieve a GFP-positive rate exceeding 95%.

### 2.6. Transfection of the miR-24 Mimic and Inhibitor

SMSCs were plated in 6-well plates and transfections were performed when the cells reached about 80% confluence. In each well, 5 μL of miR-24 mimic/inhibitor/NCm/NCi was combined with 120 μL of Opti-MEM medium (Invitrogen, Carlsbad, CA, USA), and separately, 5 μL of RNAiMAX (Invitrogen) was also mixed with 120 μL of Opti-MEM medium. These two mixtures were then combined and allowed to incubate for 10 min at room temperature (25 °C). Following this, 0.25 mL of the culture medium was replaced with an equal amount of the prepared transfection mixture. The cells were incubated for 48 h at 37 °C before further experiments were conducted.

### 2.7. RNA Extraction and Quantitative Reverse Transcription Polymerase Chain Reaction (RT-qPCR)

Total RNA from synovial tissues and SMSCs lysed using TRIzol reagent (Invitrogen) was isolated with an Eastep Total RNA Super Extraction Kit (Promega, Madison, WI, USA) according to the provided protocol. RNA concentration was determined using a Nanodrop spectrophotometer (Thermo Fisher Scientific, Waltham, MA, USA). The PrimeScript reverse-transcription system (Takara Biotechnology, Dalian, China) was used for reverse transcription. RT-qPCR was performed on a Roche LightCycler 480 System (Roche, Basel, Switzerland) utilizing an SYBR Green qPCR kit (Roche, Basel, Switzerland) to analyze the gene expression level. The 2^−ΔΔCt^ method was applied to analyze the expression levels of genes, using GAPDH as a reference for mRNA and U6 for miRNA. The primers used in the RT-qPCR are listed in Table 1.

### 2.8. Western Blot

SMSCs were lysed for 20 min using RIPA buffer (Beyotime, Shanghai, China) supplemented with 1% protease inhibitor and phosphatase inhibitor (Beyotime). The lysate was collected and centrifuged at 15,000× *g* for 15 min at 4 °C to collect the supernatant protein. Protein concentration was determined using the BCA Protein Quantitation Kit (Beyotime) following the manufacturer’s instructions. An appropriate volume of protein sample was added to the sodium dodecyl-sulfate polyacrylamide gel electrophoresis gel for protein electrophoresis, followed by transfer to a polyvinylidene fluoride (Millipore, Burlington, MA, USA) membrane. After blocking with 3% skim milk for 1 h at 25 °C, the membrane was incubated with primary antibodies (rabbit anti-phospho-p65, 1:1000 dilution [Cell Signaling Technology, Danvers, MA, USA]; rabbit anti-p65, 1:1000 dilution [Cell Signaling Technology]; rabbit anti-MARK4, 1:1000 dilution [Cell Signaling Technology]; rabbit anti-β-actin, 1:3000 dilution [Cell Signaling Technology]) overnight at 4 °C. After rinsing with PBST, secondary antibodies (IgG-HRP, 1:3000 dilution [Cell Signaling Technology]) were incubated for 1 h, and protein bands were visualized using ECL luminescent solution (Millipore, Burlington, MA, USA). Grayscale quantitative analysis of the bands was performed using ImageJ (version 1.53k; National Institutes of Health, Bethesda, MD, USA).

### 2.9. Immunofluorescence

SMSCs were seeded in laser confocal dishes and subjected to the corresponding treatments. Cells were fixed with 4% paraformaldehyde for 15 min, and 0.3% Triton-X100 was applied to disrupt the nuclear membrane. Cells were blocked with 5% skim milk at room temperature (25 °C) for one hour, followed by an overnight incubation at 4 °C with the primary antibody (rabbit anti-phospho-p65 antibody, 1:100; Cell Signaling Technology). SMSCs were then treated with fluorescent secondary antibodies (DyLight 594 AffiniPure Goat Anti-Rabbit IgG, 1:100, EarthOx, Millbrae, CA, USA) in the dark, stained with DAPI, and subsequently analyzed using confocal microscopy.

### 2.10. Cytometric Bead Array (CBA)

CBA was used to detect the IL-6 concentration in SMSC supernatant. The cell supernatant was collected and centrifuged at 3000× *g* for 10 min. The CBA kit (BD Bioscience, San Jose, CA, USA) was then used to measure the IL-6 concentration following the manufacturer’s instructions.

### 2.11. Luciferase Reporter Assay

Cells were seeded into a 96-well plate at 70% confluence. After 16 h the cells were transfected with 100 nM miR-24 mimic and miRNA mimic control. SMSCs were then co-transfected with the MARK4 3′ untranslated region (UTR) and mutated (mut) MARK4 3′ UTR luciferase vectors purchased from Heyuan (Shanghai, China). After 12 h, relative luciferase activity was tested and calculated using the Promega dual luciferase kit (Madison, WI, USA) in accordance with the manufacturer’s instructions. The wild-type or mutant MARK4 3′ UTR was positioned downstream of the firefly-luciferase coding sequence. Firefly luciferase activity was normalized to Renilla luciferase activity, and the resulting Luc/Ren ratios were used for statistical analysis. The assay was performed in three independent biological experiments.

### 2.12. Animal Experiments

All animal procedures were approved by the Animal Ethics and Welfare Committee of Sun Yat-sen University (approval no. SYSU-IACUC-2019-000144). Thirty male Sprague-Dawley rats, approximately 4 weeks of age and weighing 140–160 g, were obtained from the Center of Experimental Animals, Sun Yat-sen University. The animals were specific-pathogen-free, wild-type, experimentally naïve, and had undergone no previous procedures. Before the experiments, rats were acclimatized for 7 days in the animal facility of Sun Yat-sen University. They were housed under specific-pathogen-free conditions with controlled temperature and humidity, a 12 h light/dark cycle, free access to standard chow and water, and appropriate environmental enrichment.

Rats were allocated in a 1:1:1 ratio to three experimental groups using a computer-generated randomization sequence: the NC group, OA + NC group, and OA + miR-24 agomir group (*n* = 10 per group; 30 rats in total). The individual rat was considered the experimental unit. Cage positions were balanced, and the order of treatment administration and outcome measurements was randomized to minimize potential confounding. The investigator performing the injections was aware of group allocation, whereas outcome assessors and data analysts were blinded to group assignment.

The animal sample size of 10 rats per group was selected a priori on the basis of previous related experimental work and practical feasibility. No formal a priori power calculation was performed. The primary outcome of the animal experiment was synovial Il-6 expression. Additional outcomes included synovial expression of *Mmp13*, *Mark4*, *Col2*, *Sox9*, *Aggrecan*, and *miR-24*, as well as micro-computed tomography (micro-CT) and gross morphological assessment of the temporomandibular joint.

A priori inclusion criteria required animals to be healthy male Sprague-Dawley rats meeting the specified age and weight range. Prespecified exclusion criteria included intercurrent illness, major protocol deviation, failed OA induction, or an unusable biological sample. No animals met the exclusion criteria, and all 30 animals completed the study. Micro-CT and gross morphological assessments were performed on 10 rats per group, whereas synovial RT-qPCR analyses were performed using samples from three rats randomly selected per group. Bilateral synovial tissues from each rat were pooled and treated as one biological sample.

To induce experimental TMJ OA, rats in the OA + NC and OA + miR-24 agomir groups received bilateral intra-articular injections of 200 μL type II collagenase (20 mg/mL) into the superior joint cavity, whereas rats in the NC group received an equivalent volume of sterile normal saline using the same injection procedure. Four weeks after OA induction, rats in the NC and OA + NC groups received 100 μL of miR-24 negative control per joint, whereas rats in the OA + miR-24 agomir group received 100 μL of miR-24 agomir per joint. Intra-articular treatment was administered once every 3 days for 4 weeks. Rats were anesthetized with sodium pentobarbital during intra-articular injection procedures to minimize pain and distress.

Animals were monitored daily for general health and signs of pain or distress. Prespecified humane endpoints included body-weight loss greater than 15%, impaired feeding or mobility, and signs of infection. No animal reached a humane endpoint, and no expected or unexpected adverse events were observed during the experiment. At the experimental endpoint, animals were euthanized with an overdose of sodium pentobarbital. Bilateral TMJ specimens were collected for subsequent analyses.

The collected TMJs were examined by micro-CT, and the gross morphology of the articular disc and mandibular condyle was documented using a stereomicroscope. Synovial tissues were collected for RNA extraction and RT-qPCR analysis of *Il-6*, *Mmp13*, *Mark4*, *Col2*, *Sox9*, *Aggrecan*, and *miR-24*. RT-qPCR analyses were performed using synovial samples from three rats per group.

### 2.13. Statistical Analysis

**Figure 1:**
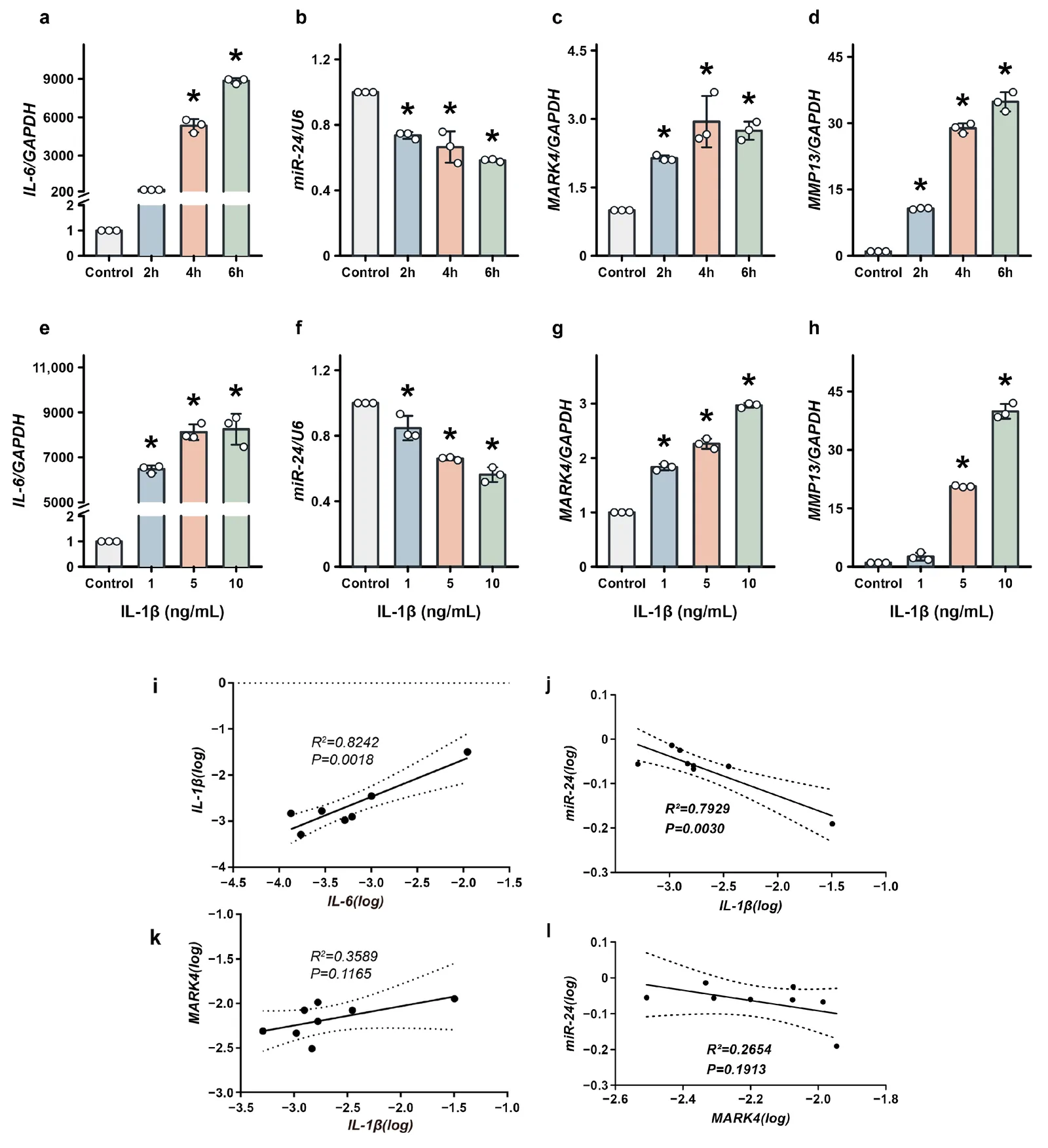
IL-1β alters miR-24 and MARK4 expression in SMSCs, with exploratory associations observed in human TMJ OA synovial tissues. (**a**–**d**) RT-qPCR was used to measure the relative expression levels of IL-6, miR-24, MARK4, and MMP-13 in SMSCs after treatment with 10 ng/mL IL-1β for 2, 4, and 6 h. (**e**–**h**) The relative expression of IL-6, miR-24, MARK4, and MMP-13 in SMSCs exposed to 1, 5, and 10 ng/mL IL-1β for 6 h was also assessed using RT-qPCR. (**i**–**l**) Correlation analysis of the expression between (**i**) IL-1β and IL-6, (**j**) miR-24 and IL-1β, (**k**) MARK4 and IL-1β, and (**l**) MARK4 and miR-24 in synovial tissues from TMJ OA patients after log transformation was performed. * *p* < 0.05 compared to the control group. For cell experiments, data are presented as mean ± SD from three independent biological experiments. Correlation analyses were performed using synovial samples from eight patients.

**Figure 2:**
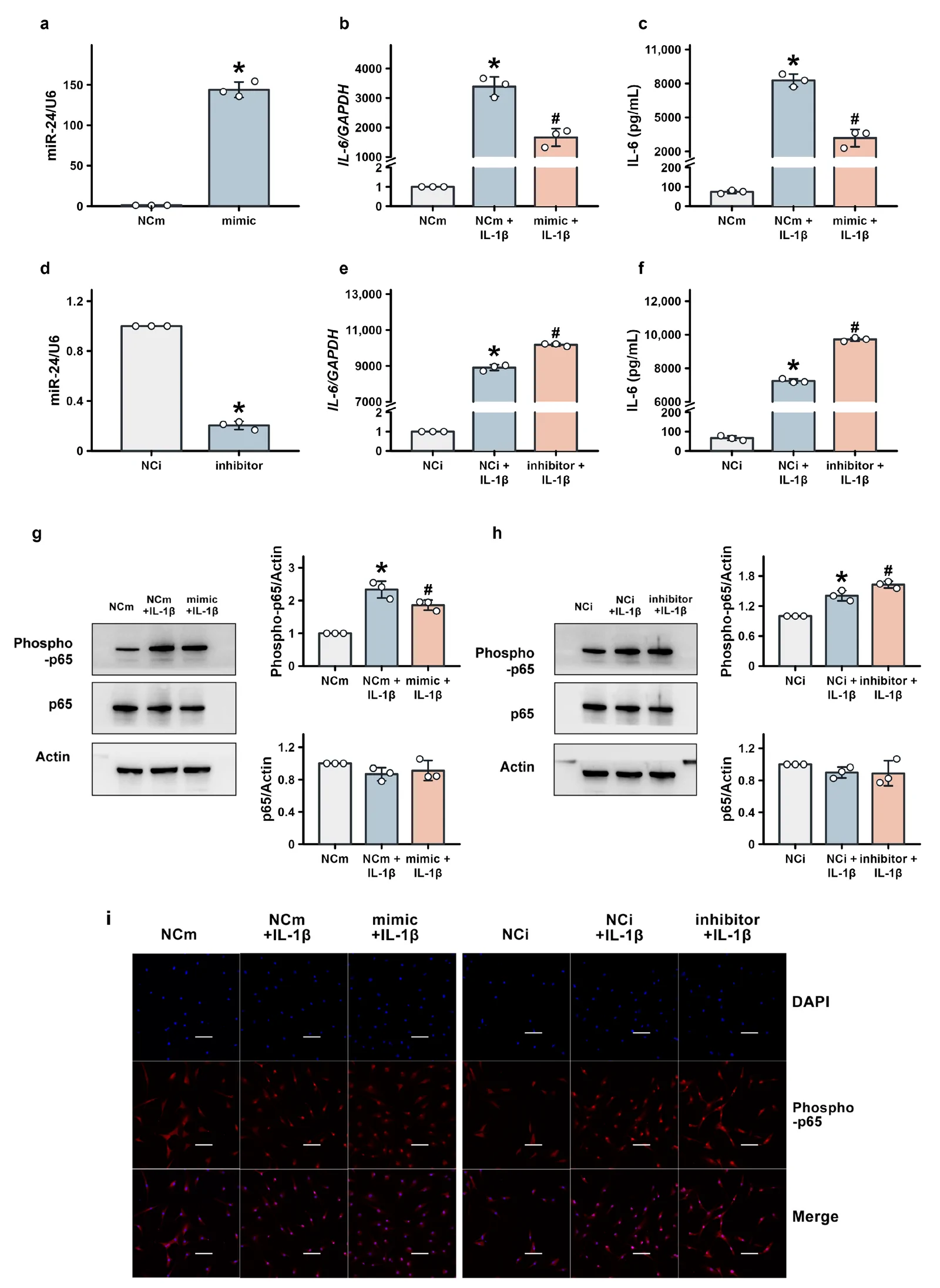
miR-24 attenuated the inflammatory activation of SMSCs via the NF-κB pathway. (**a**) Relative expression of miR-24 in SMSCs after miR-24 mimic transfections were detected using RT-qPCR. (**b**,**c**) Effects of the miR-24 mimic on the expression (**b**) and secretion (**c**) levels of IL-6 in SMSCs were detected using RT-qPCR and CBA, respectively. (**d**) Relative expression of miR-24 in SMSCs after miR-24 inhibitor transfection. (**e**,**f**) Effects of the miR-24 inhibitor on the expression (**e**) and secretion (**f**) levels of IL-6 in SMSCs were detected using RT-qPCR and CBA respectively. (**g**,**h**) Effects of the miR-24 mimic (**g**) and miR-24 inhibitor (**h**) on phospho-p65 and p65 protein levels were detected by Western blot and quantified. (**i**) Immunofluorescence analysis of the expression and localization of phospho-p65 in SMSCs with the transfection of miR-24 mimic (left) and inhibitor (right). Red fluorescence indicates phospho-p65, blue fluorescence indicates DAPI-stained nuclei, and merged images show the overlay of the two channels. * *p* < 0.05 when compared with the mimic negative controls (NCm) or inhibitor negative controls (NCi) group. # *p* < 0.05 when compared with the NCm + IL-1β or NCi + IL-1β group. Scale bars = 100 μm. The data shown represent the mean ± SD of three independent biological experiments.

**Figure 3:**
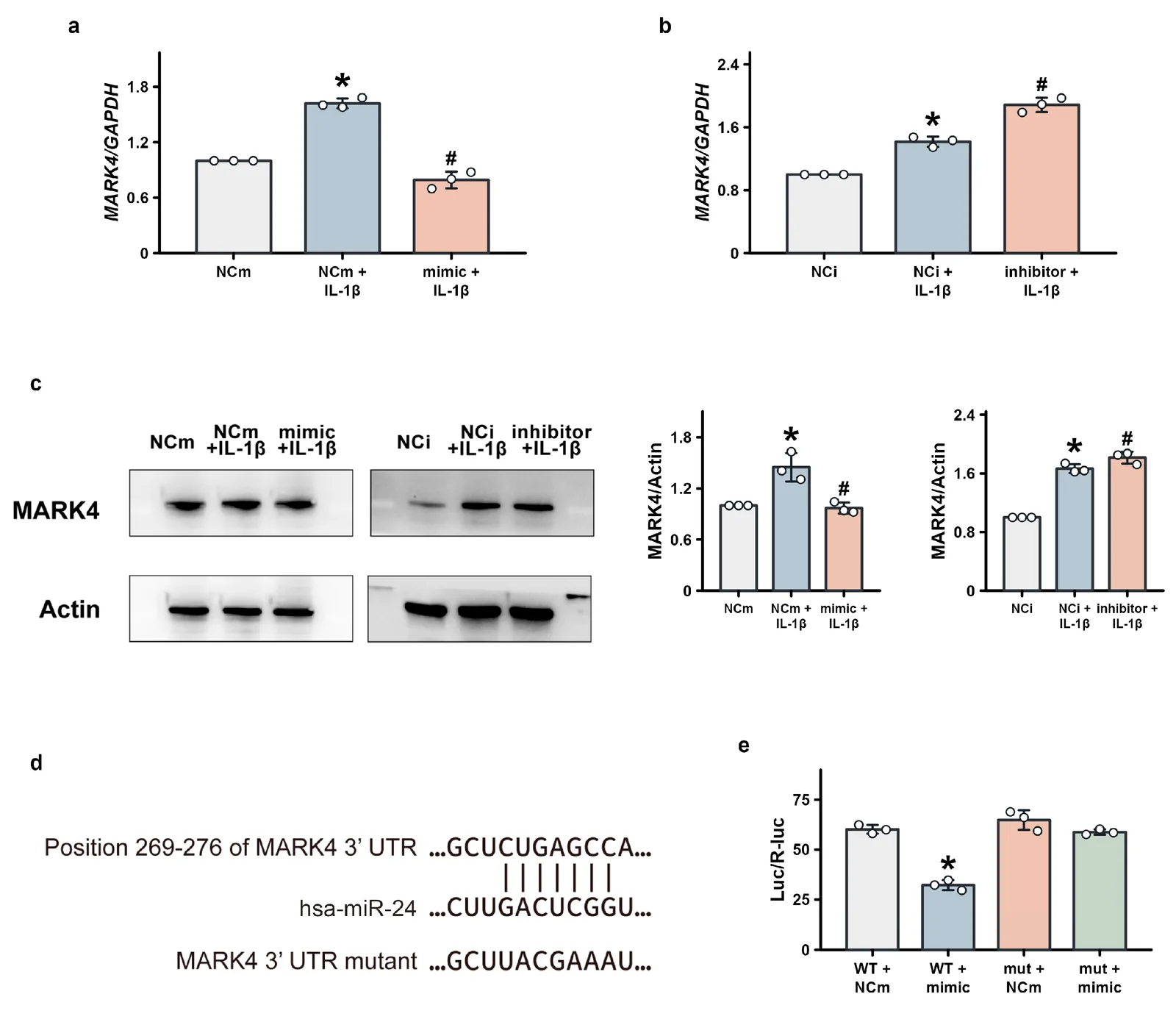
miR-24 targeted MARK4 directly. (**a**,**b**) Effects of the miR-24 mimic (**a**) and miR-24 inhibitor (**b**) on the gene expression of MARK4 were detected by RT-qPCR. * *p* < 0.05 when compared with the NCm or NCi group. # *p* < 0.05 when compared with the NCm + IL-1β or NCi + IL-1β group. (**c**) Effects of miR-24 mimic and miR-24 inhibitor on the protein level of MARK4 were detected by Western blot and quantified. * *p* < 0.05 when compared with the NCm or NCi group. # *p* < 0.05 when compared with the NCm + IL-1β or NCi + IL-1β group. (**d**) Sequence of the putative or mutated miR-24 binding site within the 3′ UTR of the MARK4 mRNA. Ellipses indicate flanking nucleotides omitted from the displayed sequences. (**e**) The MARK4 3′ UTR (WT) reporter plasmid or a mutant MARK4 3′ UTR (mut) reporter plasmid was co-transfected with NCm and miR-24 mimic into SMSCs. * *p* < 0.05 when compared with the WT + NCm group. The data shown represent the mean ± SD of three independent biological experiments.

**Figure 4:**
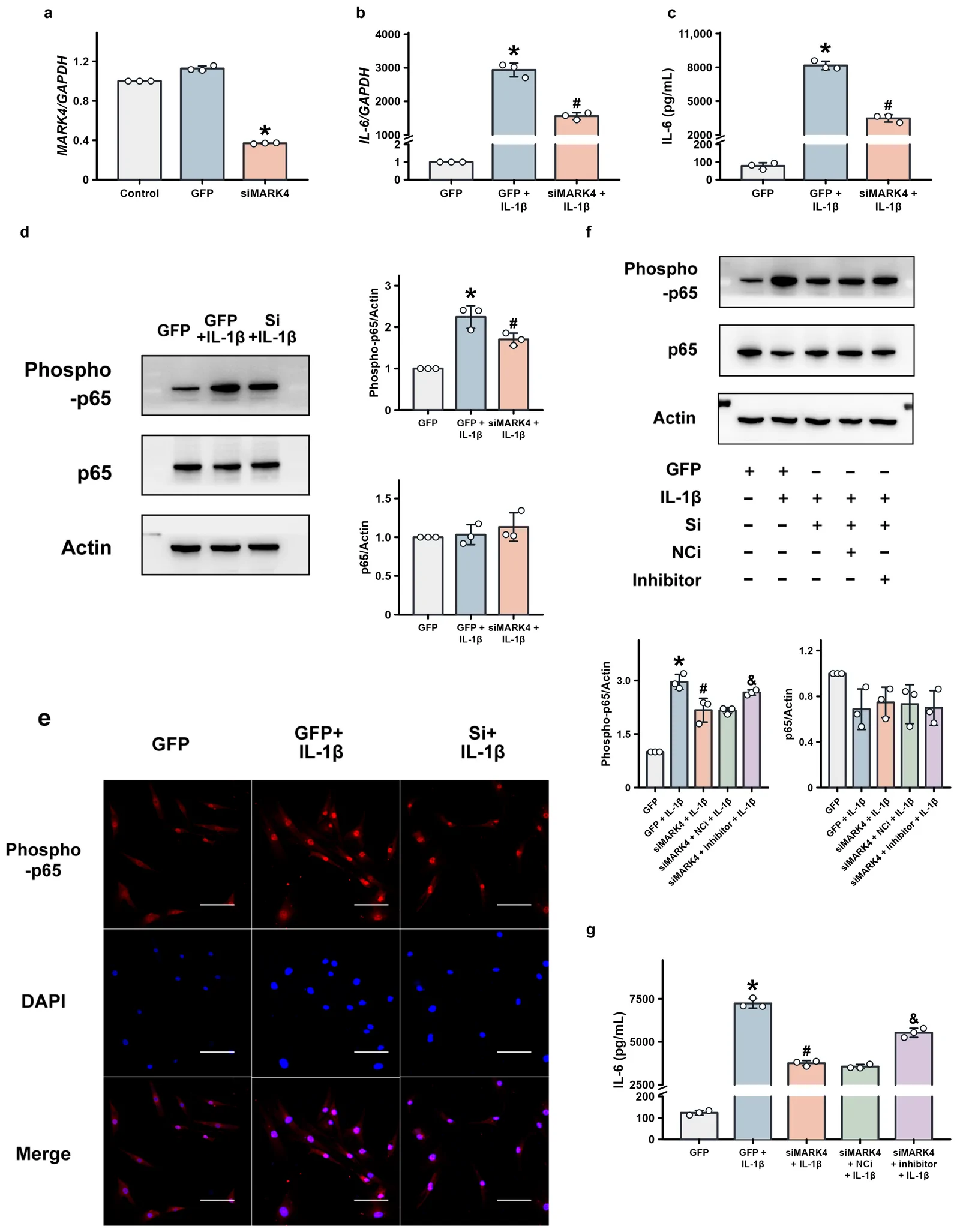
miR-24-MARK4 can regulate the expression of IL-6 in SMSCs inflamed by IL-1β via NF-κB pathway. (**a**) The relative expression of MARK4 in SMSCs following lentivirus transfection was analyzed using RT-qPCR. (**b**,**c**) The impact of siMARK4 lentivirus transfection on IL-6 expression (**b**) and secretion (**c**) levels in SMSCs was measured using RT-qPCR and CBA, respectively. (**d**) The protein levels of phospho-p65 and p65 following siMARK4 lentivirus transfection were quantified by Western blot analysis. (**e**) Immunofluorescence was used to evaluate the expression and localization of phospho-p65 in SMSCs transfected with the siMARK4 lentivirus. * *p* < 0.05 compared to the GFP group. # *p* < 0.05 compared to the GFP + IL-1β group. Scale bars = 100 μm. Data are presented as mean ± SD from three independent biological experiments. (**f**) The protein levels of phospho-p65 and p65 in the GFP, GFP + IL-1β, siMARK4 + IL-1β, siMARK4 + NCi + IL-1β, and siMARK4 + inhibitor + IL-1β groups were tested by Western blot and quantified. (**g**) The IL-6 secretion levels in the GFP, GFP + IL-1β, siMARK4 + IL-1β, siMARK4 + NCi + IL-1β, and siMARK4 + inhibitor + IL-1β groups were measured using CBA. * *p* < 0.05 compared to the GFP group. # *p* < 0.05 compared to the GFP + IL-1β group. & *p* < 0.05 compared to the siMARK4 + NCi + IL-1β group.

**Figure 5:**
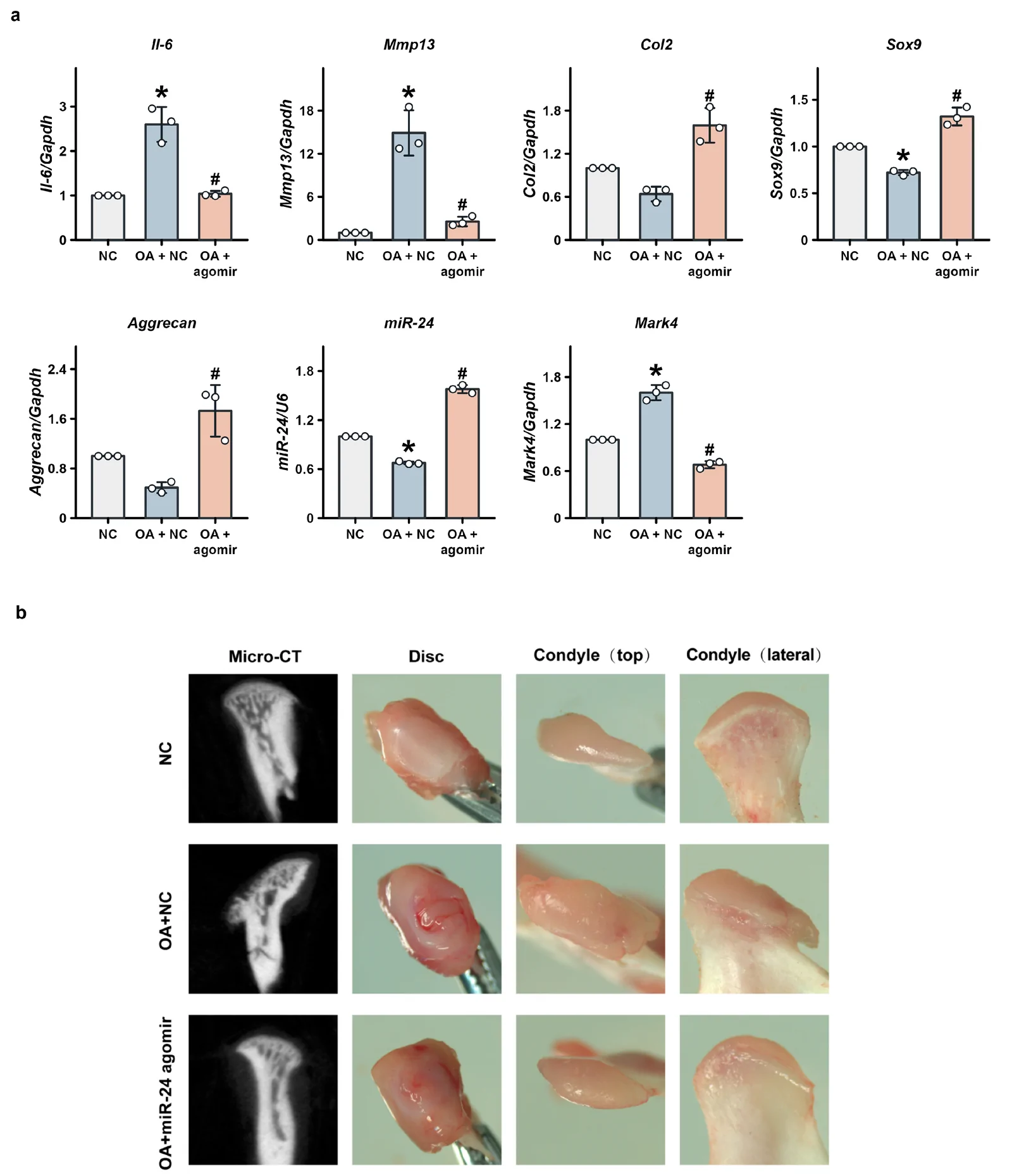
Effects of intra-articular miR-24 agomir on molecular and morphological features of experimental TMJ OA in rats. (**a**) RT-qPCR detection of Il-6, Mmp13, Mark4, miR-24, Col2, Sox9, and Aggrecan expression levels in the synovial tissues from the temporomandibular joint in rats. (**b**) Micro-CT scanning and stereo microscope imaging results of the rat temporomandibular joint. * *p* < 0.05 when compared with the NC group. # *p* < 0.05 when compared with the NC + OA group. Data are presented as mean ± SD from three biological samples per group.

Statistical analyses were performed using SPSS 22.0 software (IBM Corp., Armonk, NY, USA). Data are presented as mean ± SD. Comparisons between two groups were performed using a two-sided unpaired Student’s *t*-test. For experiments comparing multiple treatment conditions with a single control, one-way ANOVA followed by Dunnett’s multiple-comparisons test was used; other multi-group comparisons were analyzed by one-way ANOVA followed by Tukey’s HSD test. Because of the small biological sample sizes, formal normality testing was interpreted cautiously. Homogeneity of variance was assessed using Levene’s test where applicable. When variance heterogeneity was identified, Welch-based analyses were additionally performed as sensitivity analyses. Pearson correlation was used for the primary exploratory analysis of the human tissue data, with Spearman rank correlation additionally performed as a sensitivity analysis. Two-sided *p* values < 0.05 were considered statistically significant. Detailed numerical data and statistical results corresponding to Figures 1–5 are provided in Supplementary Tables S1–S22.

## 3. Results

### 3.1. IL-1β Alters miR-24 and MARK4 Expression in SMSCs and Exploratory Associations Are Observed in Human TMJ OA Synovial Tissues

SMSCs were treated with different final concentrations of IL-1β for varying periods of time. The RT-qPCR results showed that miR-24 decreased in a time-dependent and concentration-dependent manner, whereas *MARK4*, *IL-6*, and *MMP13* were upregulated (Figure 1a–h). According to the gene expression analysis in the synovial tissues of patients with OA, there was a positive correlation between *IL-6* and *IL-1β* (Pearson *r* = 0.9079, 95% CI 0.5641–0.9834, *p* = 0.0018) (Figure 1i). The expression of *miR-24* was negatively correlated with that of *IL-1β* (*r* = −0.8904, 95% CI −0.9801 to −0.4987, *p* = 0.0030) (Figure 1j). The inverse association between IL-1β and miR-24 remained significant in the Spearman sensitivity analysis (ρ = −0.8144, *p* = 0.0138), whereas the IL-1β–IL-6 association did not retain statistical significance (ρ = 0.5629, *p* = 0.1463). No statistically significant correlation was detected between MARK4 and IL-1β (*r* = 0.5991, *p* = 0.1165) (Figure 1k). For MARK4 and miR-24, the Pearson correlation was not statistically significant (*r* = −0.5152, *p* = 0.1913) (Figure 1l), whereas the Spearman sensitivity analysis yielded ρ = −0.7143 (*p* = 0.0465). Given the small sample size and the discordance between correlation methods, this finding was considered exploratory and method-sensitive. Detailed information on the patients with OA is listed in Supplementary Table S23.

### 3.2. miR-24 Attenuates the Inflammatory Activation of SMSCs by IL-1β via Inhibition of the NF-κB Pathway

IL-6 expression levels and NF-κB signaling were assessed to investigate the effects of miR-24 on inflammation activation in SMSCs treated with IL-1β. The IL-1β treatment was found to upregulate IL-6 and phospho-p65 levels, as determined by qPCR and Western blot (Figure 2b,c,e–h). The immunofluorescence also showed a notable increase in phospho-p65 in SMSCs (Figure 2i). Overexpression and downregulation of miR-24 was achieved using miR-24 mimic and inhibitor transfection respectively (Figure 2a,d). After miR-24 upregulation, IL-6 was downregulated at both the gene and protein levels, as indicated by the qPCR and CBA results (Figure 2b,c). In contrast, the downregulation of miR-24 using the miR-24 inhibitor transfection enhanced the increase in IL-6 induced by IL-1β (Figure 2e,f). Additionally, phospho-p65 was significantly downregulated compared to that in the NCm + IL-1β group after miR-24 overexpression in SMSCs (Figure 2g). Immunofluorescence also showed a decrease in phospho-p65 levels in these SMSCs (Figure 2i). Conversely, miR-24 knockdown had the opposite effect on phospho-p65 expression (Figure 2h,i). IL-1β treatment, as well as the miR-24 mimic and inhibitor, did not significantly affect p65 expression.

### 3.3. miR-24 Directly Targets MARK4

In the miR-24-overexpressing and knockdown cells, *MARK4* expression was downregulated and upregulated at the gene and protein levels, respectively (Figure 3a–c). To further elucidate the molecular mechanism by which miR-24 regulates MARK4, the sequence of the 3′ UTR from the MARK4 mRNA was analyzed using bioinformatics programs such as TargetScan (http://www.targetscan.org; accessed on 1 April 2020), miRDB (http://www.mirdb.org/; accessed on 1 April 2020), PicTar (http://pictar.mdc-berlin.de/; accessed on 1 April 2020), and miRWalk (http://zmf.umm.uni-heidelberg.de/apps/zmf/mirwalk2/; accessed on 1 April 2020). The 3′ UTR of the human MARK4 was found to contain a potential miR-24 binding site (Figure 3d). A dual luciferase assay revealed that the overexpression of miR-24 significantly reduced wild-type MARK4 luciferase activity, with no significant effect on mutants, indicating that the miR-24 reduces luciferase activity by binding to the 3′ UTR of MARK4 (Figure 3e).

### 3.4. miR-24–MARK4 Regulates IL-6 Expression in SMSCs Inflamed by IL-1β via the NF-κB Pathway

To further clarify the role of MARK4 in the activation of SMSCs inflamed by IL-1β, a lentivirus was used to knockdown MARK4 in the SMSCs. After transfection, *MARK4* expression was 30–40% of that of the control group (Figure 4a). Following MARK4 knockdown, IL-6 was downregulated at both the gene and protein levels in SMSCs stimulated with IL-1β (Figure 4b–d). Simultaneously, when MARK4 was knocked down, the expression of the phospho-p65 protein, upregulated by IL-1β, was also downregulated (Figure 4d). The expression of p65 was not affected by MARK4 knockdown. Immunofluorescence further indicated the inhibition of phospho-p65 expression (Figure 4e). To further verify whether miR-24 targets MARK4, we inhibited the effect of miR-24 following MARK4 knockdown and found that the expression of phosphorylated p65 and IL-6 was upregulated (Figure 4f,g).

### 3.5. miR-24 Agomir Modulates Molecular and Morphological Features Associated with Experimental TMJ OA in Rats

A rat model for TMJ OA was successfully constructed by injecting type II collagenase into the upper cavity of the TMJ. The RT-qPCR results showed that the expression levels of *Il-6*, *Mmp13*, and *Mark4* were upregulated, while the expression levels of *miR-24* and *Sox9* were downregulated. *Col2* and *Aggrecan* showed no statistically significant differences (Figure 5a). Stereo microscope results revealed that the articular disc in the OA group was damaged, and the articular surface was uneven; CT imaging showed that the cortical bone disappeared (Figure 5b). When compared to the OA + NC injection group, miR-24 expression was upregulated in the miR-24 agomir injection group, which was accompanied by lower expression of *Il-6*, *Mmp13*, and *Mark4* and higher expression of *Col2*, *Sox9*, and *Aggrecan* (Figure 5a). The results of the stereo microscope showed that intra-articular miR-24 agomir was associated with descriptive differences in micro-CT and gross joint morphology compared with the OA + NC group (Figure 5b).

## 4. Discussion

Recent studies have indicated that miR-24 has a crucial role in the progression of OA in MSCs [22,23]. However, the role of miR-24 in inflammation activation of SMSCs remains to be illustrated. In this study, we found that miR-24 attenuated IL-1β-induced inflammatory responses in SMSCs, with the findings supporting a contribution of MARK4 to these effects.

IL-1β has been identified as a major pro-inflammatory cytokine in OA and is currently considered one of the most important inflammatory factors in its development and progression. IL-1β can inhibit the expression of type II collagen and proteoglycan, causing an increase in MMP13 and inducing other inflammatory mediators, including IL-6 [24]. Studies have confirmed that IL-1β could inhibit the chondrogenesis of MSCs [25]. IL-6, a downstream cytokine of IL-1β, is considered another important causative factor in the pathogenesis of OA. A study showed that the level of IL-6 was positively correlated with the degree of articular cartilage damage in imaging [26]. IL-6 and IL-1β could synergistically promote the expression of MMP-1 and MMP-13, degrading the extracellular matrix of cartilage and promoting cartilage degradation. IL-6 could also inhibit the potential of mesenchymal stem cells to differentiate into cartilage [27]. In this study, a positive correlation between IL-1β and IL-6 levels in the synovial tissue of human TMJ OA was observed. Thus, it is essential to identify the regulatory mechanism of IL-6 expression.

Further analysis of gene expression in patients’ synovial tissue and SMSCs in vitro revealed that the expression level of miR-24 was negatively correlated with the expression of IL-1β. miR-24 is derived from miR-23-27-24 clusters, consisting of two paralogs: the cluster miR-23a-27a-24-2 (referred to as miR-23a, encoding mature miRNAs including miR-23a, miR-27a, miR-24) and the cluster miR-23b-27b-24-1 (referred to as miR-23b, encoding mature miRNAs including miR-23b, miR-27b, and miR-24) [28]. Mature miRNAs derived from miR-23-27-24 clusters were found to influence the progression of OA. miR-23a can inhibit the IL-17-mediated inflammatory response by targeting IKKα [29]. The down-regulation of miR-23b can aggravate inflammatory injuries in the ATDC5 cells induced by LPS by targeting PDCD4 [30]. Inhibition of miR-27a in intervertebral disc cells also inhibits inflammatory responses by inhibiting the MAPK pathway [31]. However, the effect of miRNAs on the inflammation response in SMSCs has not been fully illustrated. In this investigation, the overexpression of miR-24 was found to inhibit the upregulation of IL-6 and phosphorylated p65 in SMSCs induced by IL-1β. Our findings are consistent with previous studies showing that miR-24 can suppress inflammatory responses. Previous studies have reported anti-inflammatory effects of miR-24 in LPS-induced acute lung injury [16] and endometrial inflammation [32]. miR-24 has also been identified as an miRNA marker in the plasma of patients with rheumatoid arthritis [33]. Another study showed that miR-24 attenuated IL-1β-induced chondrocyte injury [22]. In addition, in the preclinical rat model, intra-articular miR-24 agomir was associated with reduced inflammatory gene expression and increased expression of cartilage-related genes. It has been shown that hydrogels loaded with miR-24 can inhibit senescence, improve the homeostasis of articular chondrocytes, and repair cartilage defects, which is consistent with our findings [15]. These findings were obtained in the SMSC preparations examined and do not establish cell-type specificity. Whether the miR-24–MARK4 relationship operates in other joint-resident cells remains to be determined.

We further analyzed the specific mechanism by which miR-24 regulates IL-6 expression. Using bioinformatics programs, the 3′ UTR of human MARK4 was found to contain a potential miR-24 binding site. In vitro, the expression of MARK4 in SMSCs was downregulated with miR-24 overexpression, while the knockdown of miR-24 promoted the expression of MARK4. Furthermore, rescue experiments revealed that the miR-24 inhibitors could reverse the downregulation of IL-6 and phosphorylated p65 induced by MARK4 knockdown. The dual luciferase assay also confirmed that miR-24 could directly target MARK4. Together, these findings support the involvement of the miR-24–MARK4 axis in the regulation of NF-κB-associated inflammatory responses in SMSCs. Previous studies indicate that the MARK family is regulated by various miRNAs. MARK1 was the target of miR-125a-5p to influence cell migration [34]. miR-515-5p could inhibit the proliferation of breast cancer cells by targeting MARK4 [35]. While previous studies have identified various targets of miR-24 in different contexts (e.g., BCL2L12 in chondrocytes [22] and TRAF6 in endometrial cells [32]), our study uniquely identifies MARK4 as a direct target of miR-24 in SMSCs. This difference in target specificity may be due to the distinct cellular environments and signaling pathways involved in different tissues.

The NF-κB pathway plays a crucial role in regulating reactive oxygen species production and the inflammatory response [36]. Several strategies have been implemented to suppress inflammation by inhibiting the NF-κB pathway in OA, including the use of small-molecule inhibitors, natural compounds, and gene therapy approaches. For instance, small molecule inhibitors such as BMS-345541 and SC-514 have been shown to inhibit NF-κB activation by targeting IκB kinase (IKK) activity, thereby reducing the production of pro-inflammatory cytokines [37,38]. However, the NF-κB signaling pathway includes multiple components and exerts its effects in various physiological responses, making it difficult to avoid potential side effects associated with NF-κB inhibitors [39]. Therefore, it is very important to develop disease-specific NF-κB pathway inhibitors. In this study, IL-1β was found to promote IL-6 expression by activating the NF-κB pathway. The knockdown of MARK4 and upregulation of miR-24 effectively inhibited the expression of phosphorylated p65, thereby suppressing IL-6 expression. The miR-24–MARK4 axis may represent a potential strategy for more targeted modulation of NF-κB-associated inflammation.

Members of the MARK family have a similar structure, consisting of three main regions: the N-terminal catalytic domain, the C-terminal sequence, and the ubiquitin domain [40]. Research indicates that the cell cytoskeleton may play a role in inflammation regulation [41,42]. In HL-60 monocyte-like cells, actin cytoskeleton reorganization has been shown to enhance IL-8 secretion [43]. Furthermore, MARK4 could regulate the activation of the NF-κB signaling pathway [44]. In this study, the downregulation of MARK4 was shown to inhibit IL-6 upregulation in SMSCs via the inhibition of the NF-κB pathway. Our previous study showed that knocking down MARK4 can inhibit the nuclear translocation of phosphorylated p65, while inhibition of the NF-κB pathway does not affect the expression of MARK4 [20]. Therefore, it is suggested that MARK4 may act as a critical link between cytoskeletal dynamics and pro-inflammatory signaling pathways. However, our study did not perform MARK4 overexpression experiments, which could have provided further insights into the functional consequences of MARK4 upregulation in the context of TMJ OA. Moreover, MARK4 may play a role in regulating IL-1β expression and secretion in the synovium. Studies have shown that MARK4 can bind to NLRP3 to promote the synthesis of IL-1β, and MARK4 knockdown can inhibit the expression of IL-1β [17]. Although our study did not directly investigate the role of NLRP3 in the IL-1β/MARK4 pathway, it is possible that MARK4 may also modulate NLRP3 inflammasome activation, thereby contributing to the inflammatory response in TMJ OA. Additionally, MARK4′s role in cytoskeletal dynamics may influence the secretion of IL-1β, as cytoskeletal reorganization has been shown to affect cytokine release [43]. Future studies should explore the potential crosstalk between MARK4, NLRP3, and the NF-κB pathway in the context of IL-1β-induced inflammation.

Several limitations should be acknowledged. First, the human tissue analysis was based on a small surgical cohort of eight patients and should therefore be considered exploratory. Second, although MARK4 knockdown and reporter experiments support involvement of the miR-24–MARK4 axis, complementary MARK4 overexpression experiments were not performed, and additional miR-24 targets cannot be excluded. Third, only male rats were used, limiting assessment of potential sex-related differences. Fourth, the collagenase-induced TMJ OA model does not fully reproduce the multifactorial pathogenesis of human TMJ OA. Fifth, the in vivo study primarily relied on gene-expression measurements and descriptive micro-CT/gross morphological observations without quantitative structural or histological scoring. Finally, the long-term efficacy and safety of intra-articular miR-24 administration were not evaluated. The marked miR-24 overexpression induced by mimic transfection may also not reflect physiological levels, and potential cytotoxic or nonspecific effects were not quantitatively assessed. Accordingly, the present findings should be regarded as preclinical and hypothesis-generating and do not establish the clinical efficacy or safety of intra-articular miR-24 administration in patients with TMJ OA.

## 5. Conclusions

In conclusion, miR-24 attenuated IL-1β-induced IL-6 upregulation and NF-κB activation in SMSCs, with the findings supporting a contribution of MARK4 to these responses. In a preclinical rat model, intra-articular miR-24 agomir was associated with changes in inflammatory and cartilage-related gene expression and with morphological features of experimental TMJ OA. These findings are hypothesis-generating and warrant further mechanistic, structural, safety, and translational evaluation.

## Figures and Tables

**Table 1:** Primer sequences for RT-qPCR.

Gene	Forward	Reverse
*Human GAPDH*	GACAGTCAGCCGCATCTTCT	TTAAAAGCAGCCCTGGTGAC
*Human MARK4*	CTCTTTGATTTCTTCCCGTGT	GCTATGAGGGTGAGGAGTTGA
*Human IL-6*	ACTCACCTCTTCAGAACGAATTG	CCATCTTTGGAAGGTTCAGGTTG
*Human IL-1β*	CCAGGGACAGGATATGGAGCA	TTCAACACGCAGGACAGGTACAG
*Human MMP13*	GACTGGTAATGGCATCAAGGGA	CACCGGCAAAAGCCACTTTA
*Rat Gapdh*	AGACAGCCGCATCTTCTTGT	CTCGTGGTTCACACCCATCA
*Rat Mark4*	CTCCTCGGGTTCCGTGTAT	TGCGGAGATTTCTGGTGC
*Rat Il-6*	CCGGAGAGGAGACTTCACAG	CAGAATTGCCATTGCAACAAC
*Rat Sox9*	TGAATCTCCTGGACCCCTTC	GACGTGTGGCTTGTTCTTGG
*Rat Col2*	CGGGAGCATATAACCGGAGC	CATCGCAGAGGACATTCCCA
*Rat Aggrecan*	CTTCCGCTGGTCTGATGG	TAGCCTGTGCTTGTAGGTGTTG
*Rat Mmp13*	AGACCTCCAGTTTGCAGAGCGCGT	ACCCACATCAGGAACCCCGCAT

## Data Availability

The analyzed data sets generated during the present study are available from the corresponding author on reasonable request.

## Supplementary Materials

The following supporting information can be downloaded at: https://www.mdpi.com/article/10.3390/cimb48101025/s1.

## Abbreviations

CBA: cytometric bead array; Col2: collagen type 2; DMEM: Dulbecco’s modified Eagle’s medium; GFP: green fluorescent protein; IL-6: interleukin-6; IL-1β: interleukin-1β; MARK4: microtubule affinity-regulating kinase 4; micro-CT: micro-computed tomography; miR: microRNA; MMP13: matrix metallopeptidase 13; NC: negative control; SOX9: SRY-box transcription factor 9; NCi: negative control of miR-24 inhibitor; NCm: negative control of miR-24 mimic; NLRP3: NLR family pyrin domain-containing protein 3; OA: osteoarthritis; PBS: phosphate-buffered saline; SD: standard deviation; RT-qPCR: Reverse transcription quantitative polymerase chain reaction; SMSCs: synovium-derived mesenchymal stem cells; TMJ: temporomandibular joint; UTR: untranslated region.
