# Supplementary material for: miR-24 Attenuates IL-1β-Induced IL-6 Upregulation in Temporomandibular Joint Synovium-Derived Mesenchymal Stem Cells
Source: Curr Issues Mol Biol. 2026 Oct 3;48(10):1025. doi: 10.3390/cimb48101025 (PMC13703566; doi:10.3390/cimb48101025)
Supplement: Supplementary file 1 [file cimb-48-01025-s001.zip › cimb-4552349-supplementary.pdf]

Table S1. The detailed data and statistical data of Figure 1A-1D.

gene		Control	2h	4h	6h	Control vs 2h	Control vs 4h	Control vs 6h
IL-6	mean	1	327.8	5335	8843	0.3974	<0.0001	<0.0001
	SD	0	5.709	519.3	210.8			
miR-24	mean	1	0.7356	0.6644	0.5838	0.0005	<0.0001	<0.0001
	SD	0	0.02044	0.09579	0.008396			
MARK4	mean	1	2.139	2.942	2.743	0.0042	0.0001	<0.0003
	SD	0	0.0604	0.5620	0.1992			
MMP13	mean	1	10.68	28.85	34.82	<0.0001	<0.0001	<0.0001
	SD	0	0.1702	1.108	2.171			

Table S2. The detailed data and statistical data of Figure 1E-1H.

gene		Control	1ng/mL	5ng/mL	10ng/mL	Control vs 1ng/mL	Control vs 5ng/mL	Control vs 10ng/mL
IL-6	mean	1	6473	8121	8250	<0.0001	<0.0001	<0.0001
	SD	0	168.7	346.4	686.7			
miR-24	mean	1	0.8469	0.6613	0.5624	0.0068	<0.0001	<0.0001
	SD	0	0.07463	0.009512	0.04473			
MARK4	mean	1	1.832	2.262	2.969	<0.0001	<0.0001	<0.0001
	SD	0	0.05717	0.09405	0.04271			
MMP13	mean	1	2.583	20.63	39.88	0.2448	<0.0001	<0.0001
	SD	0	1.005	0.2188	1.901			

Table S3. The detailed data and statistical data of Figure 1I-L.Pearson and Spearman correlation analyses in human TMJ OA synovial tissues.

Comparison	Pearson r	95% CI	Pearson P	R^2	Spearman ρ	Spearman P
IL-1β vs IL-6	0.9079	0.5641 to 0.9834	0.0018	0.8242	0.5629	0.1463
IL-1β vs MARK4	0.5991	−0.1827 to 0.9168	0.1165	0.3589	0.6347	0.0909
IL-1β vs miR-24	−0.8904	−0.9801 to −0.4987	0.0030	0.7929	−0.8144	0.0138
MARK4 vs miR-24	−0.5152	−0.8950 to 0.2975	0.1913	0.2654	−0.7143	0.0465

Table S4. The detailed data and statistical data of Figure 2A.

gene		NCm	mimic	NCm vs mimic
miR-24	mean	1	143.9	<0.001
	SD	0	9.508	

Table S5. The detailed data and statistical data of Figure 2B.

gene		NCm	NCm +IL-1β	mimic +IL-1β	NCm vs NCm+IL-1β	NCm+IL-1β vs mimic+IL-1β
IL-6	mean	1	3385	1664	<0.0001	0.0004
	SD	0	331.4	299.0		

Table S6. The detailed data and statistical data of Figure 2C.

protein		NCm	NCm + +IL-1β	mimic +IL-1β	NCm vs NCm+IL-1β	NCm+IL-1β vs mimic+IL-1β
IL-6	mean	74.04	8269	3175	<0.0001	<0.0001
	SD	8.654	564.5	768.5		

Table S7. The detailed data and statistical data of Figure 2D.

gene		NCi	inhibitor	NCi vs inhibitor
miR-24	mean	1	0.2045	<0.001
	SD	0	0.03312	

Table S8. The detailed data and statistical data of Figure 2E.

gene		NCi	NCi +IL-1β	inhibitor +IL-1β	NCi vs NCi+IL-1β	NCi+IL-1β vs inhibitor+IL-1β
IL-6	mean	1	8904	10179	<0.0001	<0.0001
	SD	0	162.5	81.28		

Table S9. The detailed data and statistical data of Figure 2F.

protein		NCi	NCi +IL-1β	inhibitor +IL-1β	NCi vs NCi+IL-1β	NCi+IL-1β vs inhibitor+IL-1β
IL-6	mean	66.16	7242	9716	<0.0001	<0.0001
	SD	11.82	119.0	107.0		

Table S10. The detailed data and statistical data of Figure 2G.

protein		NCm	NCm+IL-1β	mimic+IL-1β
Phospho-p65	mean	1	2.334	1.860
	SD	0	0.2572	0.1542
p65	mean	1	0.8685	0.9120
	SD	0	0.08168	0.1229

	NCm vs NCm+ IL-1β	NCm+IL-1β vs mimic+IL-1β
Phospho-p65	0.0002	0.0353
p65	0.2213	0.8124

Table S11. The detailed data and statistical data of Figure 2H.

protein		NCi	NCi+IL-1β	inhibitor+ IL-1β
Phospho-p65	mean	1	1.407	1.629
	SD	0	0.1027	0.06898
p65	mean	1	0.8999	0.8885
	SD	0	0.06893	0.1564

	NCi vs NCi+IL-1β	NCi+IL-1β vs inhibitor+IL-1β
Phospho-p65	0.0010	0.0209
p65	0.4735	0.9892

Table S12. The detailed data and statistical data of Figure 3A.

gene		NCm	NCm + +IL-1β	mimic +IL-1β	NCm vs NCm+IL-1β	NCm+IL-1β vs mimic+IL-1β
MARK4	mean	1	1.621	0.7917	<0.0001	<0.0001
	SD	0	0.05350	0.08998		

Table S13. The detailed data and statistical data of Figure 3B.

gene		NCi	NCi +IL-1β	inhibitor +IL-1β	NCi vs NCi+IL-1β	NCi+IL-1β vs inhibitor+IL-1β
MARK4	mean	1	1.418	1.885	0.0005	0.0003
	SD	0	0.06491	0.09142		

Table S14. The detailed data and statistical data of Figure 3C.

protein		NCm	NCm+IL-1β	mimic+IL-1β
MARK4	mean	1	1.450	0.9698
	SD	0	0.1664	0.06439

NCm vs NCm+ IL-1β	NCm+IL-1β vs mimic+IL-1β
0.0042	0.0030

protein		NCi	NCi+IL-1β	inhibitor+ IL-1β
MARK4	mean	1	1.667	1.816
	SD	0	0.06163	0.08152

NCi vs NCi+IL-1β	NCi+IL-1β vs inhibitor+IL-1β
<0.0001	0.0484

Table S15. The detailed data and statistical data of Figure 3E.

		WT+NC	WT+mimic	mut+NC	mut+mimic	WT+NC vs WT+mimic	WT+NC vs mut+NC	WT+NC vs mut+mimic	WT+mimic vs mut+NC	WT+mimic vs mut+mimic	mut+NC vs mut+mimic
Luc/R-luc	mean	60.11	32.1250	64.7200	58.7238	<0.0001	0.3161	0.9422	<0.0001	<0.0001	0.1513
	SD	2.144	2.53726	4.914990	1.34434						

Table S16. The detailed data and statistical data of Figure 4A.

gene		Control	GFP	SiMARK4	Control vs GFP	GFP vs SiMARK4
MARK4	mean	1	1.128	0.3694	0.0001	<0.0001
	SD	0	0.02527	0.005314		

Table S17. The detailed data and statistical data of Figure 4B.

gene		GFP	GFP+IL-1β	Si+IL-1β	GFP vs GFP+IL-1β	GFP+IL-1β vs Si+IL-1β
IL-6	mean	1	2935	1558	<0.0001	<0.0001
	SD	0	203.8	102.8		

Table S18. The detailed data and statistical data of Figure 4C.

protein		GFP	GFP+IL-1β	Si+IL-1β	GFP vs GFP+IL-1β	GFP+IL-1β vs Si+IL-1β
IL-6	mean	78.15	8160	3461	<0.0001	<0.0001
	SD	17.89	377.1	326.0		

Table S19. The detailed data and statistical data of Figure 4D.

protein		GFP	GFP+IL-1β	Si+IL-1β
Phospho-p65	mean	1	2.242	1.704
	SD	0	0.2710	0.1503
p65	mean	1	1.033	1.132
	SD	0	0.1297	0.1839

	GFP vs GFP+IL-1β	GFP+IL-1β vs Si+IL-1β
Phospho-p65	0.0004	0.0241
p65	0.9486	0.6429

Table S20. The detailed data and statistical data of Figure 4F.

protein		GFP	GFP+IL-1β	si+IL-1β	si+NCi+IL-1β	si+inhibitor +IL-1β
Phospho-p65	mean	1	2.963	2.169	2.156	2.664
	SD	0	0.2105	0.3330	0.08985	0.07049
p65	mean	1	0.6873	0.7472	0.7317	0.6983
	SD	0	0.1777	0.1328	0.1706	0.1517

	GFP vs GFP+IL-1β	GFP+IL-1β vs si+IL-1β	si+NCi+IL-1β vs si+inhibitor+IL-1β
Phospho-p65	<0.0001	0.0025	0.0426
p65	0.1256	0.9837	0.9982

Table S21. The detailed data and statistical data of Figure 4G.

protein		GFP	GFP+IL-1β	si+IL-1β	si+NCi+IL-1β	si+inhibitor +IL-1β	GFP vs GFP+IL-1β	GFP+IL-1β vs si+IL-1β	si+NCi+IL-1β vs si+inhibitor+IL-1β
IL-6	mean	123.7	7232	3752	3559	5524	<0.0001	<0.0001	<0.0001
	SD	10.76	276.4	155.3	123.8	265.7			

Table S22. The detailed data and statistical data of Figure 5A

gene		NC	NC +OA	agomir +OA	NC vs NC+OA	NC+OA vs agomir+OA
Il-6	mean	1	2.599	1.037	0.0004	0.0004
	SD	0	0.3924	0.06654		
Mmp13	mean	1	14.9	2.567	0.0002	0.0005
	SD	0	3.148	0.6610		
Col2	mean	1	0.6390	1.595	0.0592	0.0006
	SD	0	0.1015	0.2406		
Sox9	mean	1	0.7204	1.320	0.0024	<0.0001
	SD	0	0.02770	0.09551		
Aggrecan	mean	1	0.4912	1.728	0.0966	0.0020
	SD	0	0.08779	0.4152		
Mir-24	mean	1	0.6753	1.579	<0.0001	<0.0001
	SD	0	0.01784	0.04902		
Mark4	mean	1	1.601	0.6818	<0.0001	<0.0001
	SD	0	0.09657	0.04568		

Table S23. The detailed information of the eight OA patients

Temporary ID	Sex / age (years)	Surgical side and imaging findings	Past medical history	Relevant medication use	Wilkes stage
D01	Female / 25	Right TMJ; bone destruction and resorption of the right condyle, with a rough, ill-defined surface	None	None	IV
D02	Male / 20	Left TMJ; resorption and destruction of the left condyle	None	None	IV
D03	Female / 54	Left TMJ; the left condyle appeared shortened and flattened, with localized surface roughness	Thalassemia	None	V
D04	Male / 44	Left TMJ; resorption and destruction of the left condyle	None	None	IV
D05	Male / 18	Left TMJ; resorption and destruction of the left condyle	None	None	IV
D06	Male / 21	Left TMJ; resorption and destruction of the left condyle	None	None	IV
D07	Female / 65	Left TMJ; bony proliferation and surface roughness of the left condyle	Surgery for gallstone removal	None	V
D08	Female / 41	Bilateral TMJs; bone destruction and resorption of both condyles	None	None	V
